# Oxylipins as Biomarkers for Aromatase Inhibitor-Induced Arthralgia (AIA) in Breast Cancer Patients

**DOI:** 10.3390/metabo13030452

**Published:** 2023-03-20

**Authors:** Jessica A. Martinez, Betsy C. Wertheim, Denise J. Roe, Mihra S. Taljanovic, H-H. Sherry Chow, Wade Chew, Sima Ehsani, Sao Jiralerspong, Jennifer Segar, Pavani Chalasani

**Affiliations:** 1 The University of Arizona Cancer Center, Tucson, AZ 85724, USA; 2 Department of Nutritional Sciences and Wellness, University of Arizona, Tucson, AZ 85724, USA; 3 Department of Epidemiology and Biostatistics, University of Arizona, Tucson, AZ 85724, USA; 4 Department of Radiology, University of New Mexico, Albuquerque, NM 87106, USA; 5 Department of Medicine, University of Arizona, Tucson, AZ 85724, USA

**Keywords:** aromatase inhibitors, breast cancer, oxylipins, joint pain, stiffness, arthralgia

## Abstract

Aromatase inhibitor-induced arthralgia (AIA) presents a major problem for patients with breast cancer but is poorly understood. This prospective study explored the inflammatory metabolomic changes in the development of AIA. This single-arm, prospective clinical trial enrolled 28 postmenopausal women with early-stage (0–3) ER+ breast cancer starting adjuvant anastrozole. Patients completed the Breast Cancer Prevention Trial (BCPT) Symptom Checklist and the Western Ontario and McMaster Universities Arthritis Index (WOMAC) at 0, 3, and 6 months. The plasma levels of four polyunsaturated fatty acids (PUFAs) and 48 oxylipins were quantified at each timepoint. The subscores for WOMAC-pain and stiffness as well as BCPT-total, hot flash, and musculoskeletal pain significantly increased from baseline to 6 months (all *p* < 0.05). PUFA and oxylipin levels were stable over time. The baseline levels of 8-HETE were positively associated with worsening BCPT-total, BCPT-hot flash, BCPT-musculoskeletal pain, WOMAC-pain, and WOMAC- stiffness at 6 months (all *p* < 0.05). Both 9-HOTrE and 13(S)-HOTrE were related to worsening hot flash, and 5-HETE was related to worsening stiffness (all *p* < 0.05). This is the first study to prospectively characterize oxylipin and PUFA levels in patients with breast cancer starting adjuvant anastrozole. The oxylipin 8-HETE should be investigated further as a potential biomarker for AIA.

## 1. Introduction

Adjuvant aromatase inhibitors (AIs) are the recommended endocrine treatment for postmenopausal women diagnosed with early-stage, estrogen receptor-positive (ER+) breast cancer. AIs are also used in premenopausal women in combination with gonadotropin-releasing hormone agonists (GnRH). The three third-generation AIs in routine clinical use—anastrozole, letrozole, and exemestane—have similar efficacy and toxicity profiles when compared across studies. The standard recommended duration was five years until recent clinical trials showed that extended therapy (10 years) improves the disease-free survival rate in patients with high-risk ER+ breast cancer [1]. Despite these benefits, adherence remains a challenge, as AI therapy is associated with significant, activity-limiting musculoskeletal symptoms, including arthralgia, myalgia, and joint stiffness, collectively called AI-induced arthralgia (AIA). Symptoms can manifest early after the initiation of AI therapy and worsen up to two years. High rates of AI non-adherence (estimated at up to 50% by year three) due to an intolerance to the side effects, notably AIA, are now linked to a reduced benefit [2,3]. The majority of pharmacological and non-pharmacological intervention studies for AIA are negative. This is most likely due to the enrolment of patients who are on adjuvant AIs rather than the enrolment of those “at high risk” for developing AIA. Currently, there is a need for clinically validated biomarkers to predict who is at risk for AIA, explain AIA progression, and guide intervention studies to improve quality of life and reduce death from breast cancer by improving AI adherence.

While AIA is a well-known problem, the mechanism for its development is not well understood and grossly understudied. AIs block peripheral estrogen synthesis, thereby further decreasing estrogen levels [4]. Preclinical data suggest that AIA is directly related to loss of the anti-nociceptive action of estradiol; however, the level of estradiol depletion is not correlated with the degree of AIA symptoms [5]. Inflammation also plays a role in exacerbating AIA symptoms [6], and non-steroidal anti-inflammatory drugs (NSAIDs) provide relief for some women with AIA [7]. Our group previously reported that, in patients with breast cancer who are stable on an AI, intervention with the NSAID sulindac for six months resulted in improved pain, stiffness, and physical function as assessed by the Western Ontario and McMaster Universities Osteoarthritis (WOMAC) Index [8]. However, inflammation alone is unlikely to be the cause of AIA [9]. To date, there are no clear metabolic pathways identified to explain AIA etiology or to determine targets for interventions.

Oxylipins are produced via metabolism of ω-6 and ω-3 polyunsaturated fatty acids (PUFAs) by cyclooxygenase (COX), lipoxygenase (LOX), and cytochrome P450 (CYP450) enzymes. NSAIDs target COX, which metabolizes ω-6 and ω-3 PUFA to inflammatory prostaglandins [10]. Oxylipins have a spectrum of biological activity, including pro- and anti-inflammatory effects, as well as the induction and inhibition of pain. The presence of underlying inflammation and the nociceptive activity of oxylipins, may be a contributing factor for pain [11,12,13]. Our group previously published an overview of the oxylipin pathway and biological outcomes [14]. Oxylipin profiles are implicated in the development of inflammatory conditions including rheumatoid arthritis [15]. Preliminary evidence also implicates both the CYP450 and LOX pathways in the development of tendinopathy [16,17]. We previously published that tendon stiffness may play a role in the pain experienced by women taking AIs [18,19]. AIs are also involved in the upregulation of the CYP450 pathway [20] and cross-talk between the estrogen receptor (ER) and LOX-mediated oxylipins [21], suggesting a role of oxylipins in AIA. These findings suggest that the development or progression of AIA is likely attributed, in part, to an unfavorable oxylipin profile. Alterations in the entire oxylipin cascade that result in multiple biological effects, including inflammation, the development of tendon stiffness, and increased nociception [22], may play a role in the development of AIA in patients with breast cancer.

We conducted a prospective study to further explore these inflammatory metabolomic changes in the development of AIA. Women were enrolled after the completion of their definitive treatment at the initiation of their AI and were followed for six months. We previously reported in a subgroup of these patients that baseline stiffness in the abductor pollicis longus tendon evaluated using shear wave elastography could be used to predict the development of AIA [19]. Here we report the blood-based inflammatory biomarkers evaluated in these patients. To the best of our knowledge, this is the first study to report an inflammatory profile at baseline and the changes while on AI therapy.

## 2. Materials and Methods

### 2.1. Study Design

**Figure 1:**
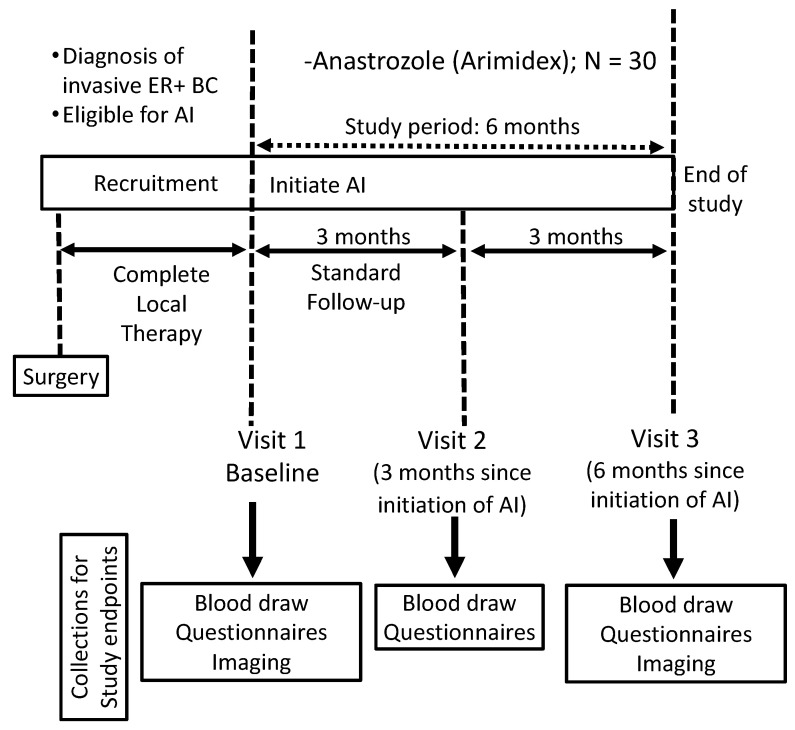
Clinical trial study design. Abbreviations. AI: Aromatase Inhibitor; BC: Breast Cancer; ER+: Estrogen Receptor Positive.

This single-arm, prospective clinical trial was conducted at the University of Arizona Cancer Center (NCT03665077). It was approved by the institutional review board, and all patients enrolled signed an informed consent. Postmenopausal women with early-stage (0–3) ER+ breast cancer who were candidates for adjuvant AI therapy and had completed their definitive treatment (surgery ± radiation) were enrolled into this study. The exclusion criteria included having received chemotherapy (adjuvant or neo-adjuvant), prior endocrine therapy (AI or tamoxifen), history of rheumatoid arthritis or other autoimmune arthritis, active daily NSAID use (other than low-dose aspirin), and active use of any corticosteroids or immunosuppressive therapies. Participants were recruited during their initial visit with their oncologist prior to the initiation of AI therapy. They completed blood draws and questionnaires at 0, 3, and 6 months after initiating AI therapy (Figure 1). To decrease confounding effects, adjuvant AI therapy was initiated 6 weeks after the completion of their definitive treatment. All women enrolled in this study were started on adjuvant anastrozole.

### 2.2. Arthralgia and Depression Outcome Measures

BCPT: The Breast Cancer Prevention Trial (BCPT) Symptom Checklist is a 42-item questionnaire validated in breast cancer survivors [23]. The BCPT total score comprises 8 subscores: hot flash (3 questions), nausea (3 questions), bladder control (2 questions), vaginal problems (3 questions), musculoskeletal pain (3 questions), cognitive problems (4 questions), weight problems (4 questions), and arm problems (2 questions). For each question, women indicate the presence or absence of symptoms and the extent to which they are bothered by those symptoms on a five-point Likert scale ranging from 0 (not at all) to 4 (extremely). The musculoskeletal pain (MS) subscale has been shown to be responsive to changes in AIA and is calculated as the mean of the responses to three questions addressing general aches and pains, joint pain, and muscle stiffness [24]. In the case of missing data for any question within the subscales, the entire subscale was considered missing.

WOMAC: The WOMAC is a 24-item instrument developed to assess pain (5 items), stiffness (2 items), and physical function (17 items) in participants with hip and/or knee osteoarthritis as well as for AIA [25,26]. Here, we evaluated the 3 subscales and the total score using the 5-point Likert format (0 = none to 4 = extreme). As discussed in Bellamy [25], for convenience and for comparison purposes to previous studies, the total scores and each subscale were normalized to a range of 0–100. In the case of missing data, the subscales were considered valid as long as no more than 1 item was missing for pain or stiffness, and no more than 4 items were missing for physical function.

Patient Health Questionnaire (PHQ)-9: The Patient Health Questionnaire (PHQ)-9 is a validated multipurpose tool used for screening, diagnosing, monitoring, and measuring the severity of depression [27]. It has been reported that up to 50% of newly diagnosed patients with breast cancer have symptoms of depression or anxiety [28], and the perception of pain may be altered in individuals with symptoms of depression [29]. The PHQ-9 is a 9-item questionnaire that asks how often a person has been bothered by symptoms within the past 2 weeks. Responses are measured on a 4-point Likert scale, including “0 = not at all”, “1 = several days”, “2 = more than half of the days”, and “3 = nearly every day”.

### 2.3. Plasma Sample Collection and Preparation

At collection, triphenylphosphine (TPP) and butylated hydroxytoluene (BHT 0.2% *w/w*) (MilliporeSigma, Burlington, MA, USA) were added to plasma that was collected in EDTA tubes. TPP reduces peroxides to their monohydroxy equivalents, and BHT quenches radical catalyzed reactions [30]. Both reagents prevent peroxyl radical propagated transformations of fatty acids. Three 300 µL aliquots of plasma plus antioxidant were frozen immediately at −80 °C. Based on our experience, oxylipins are not stable through multiple freeze-thaw cycles. Therefore, all samples were thawed only once for batch analysis.

Plasma samples were prepared for ultra-performance liquid chromatography (UPLC–MS) analysis as described in detail by Liu et al. [31]. Briefly, 250 µL plasma was spiked with a set of odd chain length analogues and deuterated isomers of several target analytes, including hydroxyeicosatetraenoic acids, thromboxanes, epoxides, prostaglandins, and diols, contained in 10 µL methanol (Cayman Chemical, Ann Arbor, MI, USA). Samples were then subjected to solid phase extraction using Oasis Prime HLB 3 mL, 60 mg sorbent (Waters, Milford, MA, USA). Eluents were evaporated to dryness and reconstituted in 50 µL methanol. Spiked samples were then vortexed, centrifuged, and transferred to autosampler vials for analysis.

### 2.4. Reverse Phase Chromatography with UPLC–MS

Oxylipin profiling was performed using UPLC with an Agilent Ultivo QQQ MS system coupled to an Agilent 1290 Infinity II UPLC system (Agilent, Santa Clara, CA, USA). Chromatographic separation of the oxylipins was achieved using a gradient of water, methanol, and acetonitrile, all with 0.1% acetic acid (*v/v*). The acquisition parameters were as previously described [32] with minor modifications, and the MS data were used for quantification. Surrogate analytes and internal and external standards were used to monitor extraction efficiency and ensure accurate quantitation with standard curves. The acquired data were quantified using Quant-My-Way (Agilent, Santa Clara, CA, USA) using 9 isotope-labeled internal standards. Here we report the data for oxylipins with >80% of values above the limit of detection (43 of 62 oxylipins) and for 4 PUFAs: arachidonic acid (ARA), linoleic acid (LA), eicosapentaenoic acid (EPA), and docosahexaenoic acid (DHA) (Cayman Chemical, Ann Arbor, MI, USA). UPLC was performed in 2 separate batches, ensuring that repeat measures across time for the same participant were all included in the same batch.

### 2.5. Statistical Analysis

Baseline characteristics were summarized using the median [interquartile range (IQR)] for continuous variables and proportions for categorical variables. The symptom scores and oxylipin levels were summarized at each time point using the mean ± standard deviation (SD). The associations between the baseline PUFA/oxylipin levels and baseline symptom scores were tested using Spearman correlations. The changes in symptom scores across time were tested using linear mixed-effects models with time (interval since baseline) as a continuous variable, adjusted for baseline symptom score, and clustered on the participant. Additional models further adjusted for age at baseline, BMI at baseline, and definitive therapy (mastectomy versus lumpectomy). Similar mixed-effects models were constructed for changes in PUFAs and oxylipins across time and adjusted for baseline level and batch. The associations between the baseline PUFA/oxylipin levels and symptom scores across time were tested using linear mixed-effects models as described above. PUFAs and oxylipins were log-transformed in all models. The statistical analyses were conducted using Stata 17.0 (StataCorp, College Station, TX, USA), and no adjustments were made for multiple comparisons.

## 3. Results

### 3.1. Participants and Characteristics

Of the 30 patients recruited, one was ineligible due to prior therapies, and one withdrew on the same day as enrollment per difficulty with the blood draw, thus yielding a sample size of 28. The median (IQR) age was 66.0 (63.1–72.6) years at enrollment (Table 1). Median (IQR) time since diagnosis was 4.7 (3.6–5.9) months. Median (IQR) BMI was 25.1 (23.0–31.3) kg/m^2^, and the cohort was 89.3% non-Hispanic white. For their definitive breast surgery, 78.6% received a lumpectomy, and 67.9% required radiation. There were eight (28.6%) participants with stage 0 breast cancer, seventeen (60.7%) stage I, and three (10.7%) stage II. There were eight participants regularly taking low-dose aspirin (81 mg) and one participant taking other (non-NSAID) pain medication.

### 3.2. Change in Symptom Scores

BCPT, WOMAC, and PHQ-9 mean *±* SD scores at baseline, three, and six months are presented in Table 2. In the fully adjusted model, there was a significant increase in the BCPT-total score (*p* = 0.008) and BCPT-MS subscore (*p* < 0.001) by six months. The BCPT-MS subscale has been shown to be responsive to changes in AIA with scores > 1.5, indicating clinically relevant arthralgia [24,33]. At baseline, there were five of 28 (18%) women with a score > 1.5 on the BCPT-MS subscale, seven of 22 (32%) at three months, and nine of 24 (38%) at six months. The BCPT-hot flash subscore also significantly increased by six months (*p* = 0.005). There was no change in the other BCPT subscores (nausea, bladder control, vaginal problems, cognitive problems, weight problems, and arm problems) across the 6-month study period.

WOMAC-pain significantly increased across time (*p* = 0.047); however, only eight of 24 women experienced a worsening of their symptoms. The mean ± SD change in the pain score for these eight women was 18.8 ± 7.9. WOMAC-stiffness also significantly increased (*p* = 0.031), which was driven by 11 of 24 women who experienced a worsening of their symptoms (27.3 ± 12.3 point change from baseline to six months for those 11 participants). Changes in the physical function subscore or the total score were not statistically significant. However, 14 women experienced a worsening of the physical function subscore (9.7 ± 8.0 point change from baseline to six months), and 14 women experienced a worsening of the WOMAC-total score (10.2 ± 8.2 point change from baseline to six months). There were no significant changes in the PHQ-9 total score for depression.

### 3.3. Correlation between Oxylipins and Symptom Scores at Baseline

Plasma samples were not available for three participants, thus yielding a sample size of 25 for these analyses. There were four PUFAs (EPA, DHA, ARA, and LA) plus 62 of their oxygenated lipid metabolites (oxylipins) in the original analytical platform. Of the 62 oxylipins, 43 had >80% of samples with levels above the limit of detection [14]. Table S1 shows the mean ± SD at baseline, three, and six months for the four PUFAs that were quantified, and Table S2 shows the mean ± SD at baseline, three, and six months for the 43 oxylipins. To characterize the relationship between the oxylipins and symptoms, the oxylipin and PUFA levels in the plasma were correlated with the symptom scores at baseline. There were no significant correlations between any oxylipins or PUFAs and the BCPT-total score or the BCPT-hot flash, BCPT-MS, or BCPT-cognitive subscores. Significantly correlated oxylipins with BCPT subscores are as follows: nausea with 9-OxoODE (ρ = 0.41; *p* = 0.041), bladder control with 8(9)-EET (ρ = 0.42; *p* = 0.037), vaginal problems with 5(6)-DiHET (ρ = 0.46; *p* = 0.024), weight problems with 8-HETE (ρ = 0.42; *p* = 0.039) and 15-HETE (ρ = 0.54; *p* = 0.005), and arm problems were negatively correlated with 13(14)-EpDPA (ρ = −0.45; *p* = 0.024), 16(17)-EpDPA (ρ = −0.41; *p* = 0.042), and 19(20)-EpDPA (ρ = −0.41; *p* = 0.042). The PHQ-9 total score was significantly correlated with 9-OxoODE (ρ = 0.50; *p* = 0.010) and negatively correlated with 8(9)-EpETE (ρ = −0.41; *p* = 0.039). There were no significant correlations between the WOMAC-total, stiffness, physical function, or pain subscores and any oxylipins or PUFAs (data not shown).

### 3.4. Change in PUFAs and Oxylipins

PUFA levels were stable across time (Table S1). Two EPA products, 8(9)-EpETE and 8(15)-DiHETE, significantly increased from baseline to six months (both *p* < 0.05). There were no significant changes in any other oxylipins (Table S2).

### 3.5. Baseline Oxylipins Predict Changes in Symptom Scores

Baseline PUFAs and oxylipins were individually included in the mixed models to test their association with symptom scores across time. No PUFAs were significantly associated with any symptom scores across time. The ARA metabolite derived from 15-LOX, 8-HETE, was positively associated with worsening BCPT-total (*p* = 0.017), BCPT-hot flash (*p* = 0.007), BCPT-MS (*p* = 0.018), WOMAC-pain (*p* = 0.001), and WOMAC-stiffness (*p* = 0.049). Two LOX-derived metabolites from alpha linolenic acid were significantly related to worsening BCPT-hot flash: 9-HOTrE (*p* = 0.005) and 13(S)-HOTrE (*p* = 0.025). The 5-LOX metabolite of arachidonic acid, 5-HETE, was also significantly related to worsening WOMAC-stiffness (*p* = 0.026).

**Figure 2:**
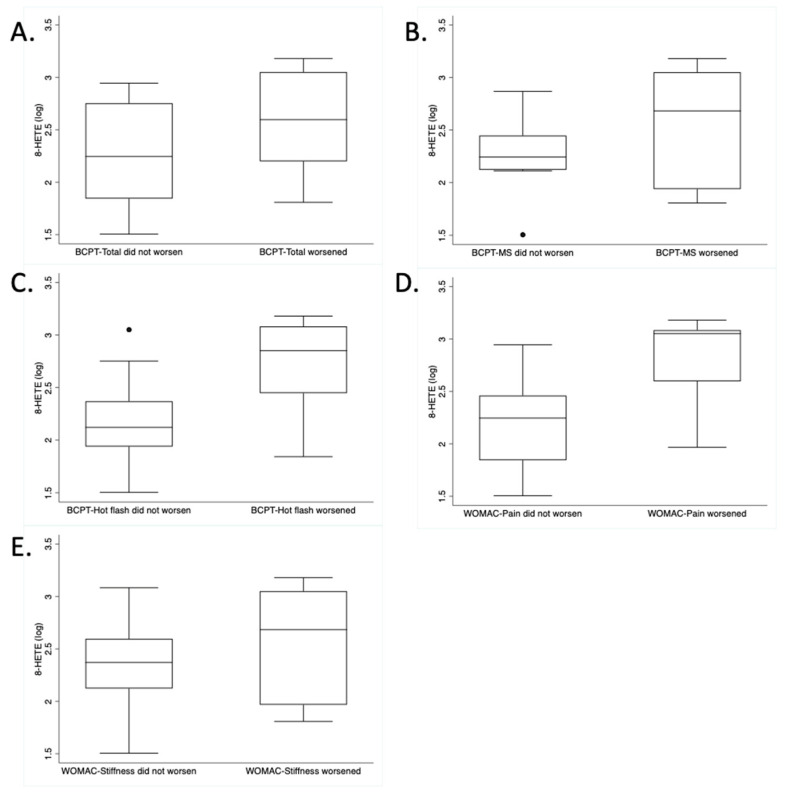
Box plots of batch-adjusted, log-transformed 8-HETE measures among the participants who did and did not experience worsening symptoms over 6 months, according to 5 different questionnaire subscales: (**A**) BCPT-total, (**B**) BCPT-MS, (**C**) BCPT-hot flash, (**D**) WOMAC-pain, and (**E**) WOMAC-stiffness. In separate linear mixed-effects models, 8-HETE was significantly associated with each of these 5 subscales across time (all *p* < 0.05).

Given that 8-HETE was the only oxylipin related to several AIA outcomes, Figure 2 illustrates the box plots comparing the baseline batch-adjusted 8-HETE measures among the participants who did and did not experience worsening symptoms (BCPT total, BCPT hot flash, BCPT-MS, WOMAC-pain, and WOMAC-stiffness) over six months. All scores were higher at baseline among those women that went on to have worsening symptoms by six months.

## 4. Discussion

The primary purpose of this study was to determine whether any baseline oxylipins or PUFAs could predict who might develop symptoms related to AIA. In this preliminary study, we found that baseline levels of 8-HETE were significantly related to worsening symptoms of AIA from baseline to six months of adjuvant therapy with anastrozole. 8-HETE is produced primarily from arachidonic acid via 15-LOX [34]. Early work showed that 8-HETE is a strong activator of peroxisome proliferator-activated receptor (PPAR) alpha and a weak activator of PPAR gamma, regulators of lipid homeostasis [35], and induces differentiation of preadipocytes [36]. Compounds that induce differentiation of adipocytes have been shown to inhibit aromatase expression and, thus, estrogen synthesis by adipose tissue [37]. To our knowledge, no studies have yet determined whether there is a relationship between 8-HETE and estrogen levels in circulation or in tissues. Another study showed that 8-HETE levels were higher in patients that had experienced a myocardial infarction relative to matched controls, and 8-HETE was significantly positively correlated with the pro-inflammatory cytokine tumor necrosis factor-alpha (TNF-α) [38]. In cell culture, 12/15-LOX overexpression has been directly linked to increased TNF-α production. These data taken together suggest the possibility that additional suppression of estrogen via 8-HETE as well as an inflammatory profile related to overexpression of 15-LOX (and thus 8-HETE production) predisposes women to AIA and explains the relationship between baseline 8-HETE and AIA development observed in our study.

In addition to 8-HETE, two LA metabolites produced via the LOX pathway, 13(S)-HOTrE and 9-OxoODE, as well as the α-LA metabolite 9-HOTrE also produced via LOX were all significantly related to the development of hot flashes by six months. To our knowledge, this is the first study to show an association between these oxylipins and hot flashes. Along with other oxidized LA metabolites, 9-OxoODE has been shown to induce nociceptive hypersensitivity in a rat model [22]. Other LOX products of LA, HODEs, have previously been shown to have pro-nociceptive properties in rodent pain behavioral models [39,40,41] and to be involved in inflammatory pain [42] and Achilles tendinopathy [16]. However, in the current study, there was no association between these LOX metabolites and pain scores on treatment with anastrazole.

We also noted that four CYP450 metabolites of DHA, all epoxydocasapentaenoic acids [7(8)-EpDPA, 13(14)-EpDPA, 16(17)-EpDPA, and 19(20)-EpDPA)], were significantly negatively correlated with arm pain as assessed with the BCPT arm subscore. To our knowledge, this is the first report to suggest an association between epoxydocasapentaenoic acids and pain. However, very few studies have investigated the relationship between these four epoxydocasapentaenoic acids and clinical outcomes. One clinical trial showed that they are elevated in hemodialysis patients [43]. Preclinical studies have shown that 19(20)-EpDPA increases the browning of white adipose tissue through the GPR120-AMPK signaling pathway [44]. ω-3 PUFAs have been shown to reduce inflammation through GPR120 [45]. Further, 19(20)-EpDPA is a potent vasodilator in microcirculatory vessels [46], and vasodilators have been shown to reduce different types of pain, including neuropathic [47,48]. Thus, women with higher circulating levels of epoxydocasapentaenoic acids may have reduced inflammation and increased vasodilation, which may explain the negative correlation with arm pain in the present study.

Interestingly, none of the PUFAs were associated with any symptom scores. Diets with a high ω-6:ω-3 ratio, associated with a Westernized eating pattern, have been associated with increased inflammatory profiles [49]. Conversely, diets rich in EPA and DHA have been associated with reduced pain and inflammation [50]. One study showed in a rat model that ω-6 fatty acids increased nociception related to nerve damage not inflammation, and dietary replacement with ω-3 PUFAs reverted the phenotype [51]. Here, the overall cohort had a 13.5:1 ω-6:ω-3 ratio, similar to the commonly reported 16:1 ratio seen in populations that consume a Western diet. Studies have shown that ratios below 5:1 are needed to have a beneficial effect on disease risk, and suppression of inflammation in rheumatoid arthritis patients was achieved at 3:1 [52]. In the current study, only two participants had an ω-6:ω-3 ratio less than 5:1. When comparing the ratios of the women that developed any symptoms relative to the women that did not in this study, there was no difference. One study in women on AI showed that supplementation with an ω-3 PUFA significantly reduced AIA; however, the reduction in pain was not different than that in the placebo [53]. Our study suggests that, while the presence of ω-3s is important, the underlying metabolism of PUFA may play a more profound role in the development of AIA, and more targeted prevention may be necessary, such as dual COX and LOX pathway inhibitors.

We also sought to characterize the change in oxylipins over time with anastrozole treatment. Overall, PUFAs and oxylipins did not change in the patients with breast cancer in response to administration of the AI anastrozole. Two EPA products, 8(9)-EpETE from the CYP450 pathway and 8(15)-DiHETE that is an sEH product, significantly increased from baseline to six months; however, given the large number of statistical tests and the lack of relationship of these oxylipins with pain outcomes, we cannot render any conclusions. To the best of our knowledge, this is the first study to prospectively characterize oxylipin and PUFA levels in women who started adjuvant anastrozole.

Our study also contributes to the literature by prospective longitudinal assessment of AIA symptoms with validated questionnaires over six months. The major limitations of our study are the small sample size and small proportion that developed symptoms of AIA, which limited our ability to interpret any changes in metabolomic profiles. Nonetheless, we are able to contribute data on baseline oxylipin and PUFA profiles in postmenopausal women, which should be explored in larger studies.

## 5. Conclusions

In conclusion, we found that the baseline level of the 15-LOX product of AA, 8-HETE, was related to worsening of several AIA symptoms. Epoxydocasapentaenoic acids may also play a role given their anti-inflammatory and vasodilating effects. Future studies should investigate 15-LOX and/or CYP450 as potential targetable pathways for AIA management.

## Figures and Tables

**Table 1:** Baseline characteristics (*n* = 28).

Characteristic	Median (IQR) or *n* (%)
Age at enrollment (y)	66.0 (63.1–72.6)
Age at diagnosis (y)	65.6 (62.7–72.3)
Time since diagnosis (months)	4.7 (3.6–5.9)
BMI (kg/m^2^)	25.1 (23.0–31.3)
Race/ethnicity	
Non-Hispanic white	25 (89.3%)
Hispanic	3 (10.7%)
Definitive breast surgery	
Mastectomy	6 (21.4%)
Lumpectomy	22 (78.6%)
Radiation	
No	9 (32.1%)
Yes	19 (67.9%)
Disease stage	
0	8 (28.6%)
I	17 (60.7%)
II	3 (10.7%)
Aspirin use (low dose)	
No	20 (71.4%)
Yes	8 (28.6%)
Pain medication (non-NSAID)	
No	27 (96.4%)
Yes	1 (3.6%)

**Table 2:** Symptom scores across time, mean ± SD.

Subscore	Baseline *n* = 28	3 Months *n* = 22	6 Months *n* = 24	*p* Crude ^a^	*p* adj-1 ^b^	*p* adj-2 ^c^
BCPT						
Total	0.53 ± 0.41	0.57 ± 0.47	0.64 ± 0.52	**0.005**	**0.009**	**0.008**
Hot flash	0.45 ± 0.81	0.91 ± 0.91	0.98 ± 1.23	**0.004**	**0.006**	**0.005**
Nausea	0.16 ± 0.31	0.30 ± 0.50	0.19 ± 0.48	0.831	0.873	0.864
Bladder control	0.59 ± 0.89	0.34 ± 0.47	0.35 ± 0.62	0.342	0.332	0.321
Vaginal problems	0.72 ± 0.84	0.62 ± 0.85	0.67 ± 1.09	0.437	0.442	0.454
Musculoskeletal pain	0.81 ± 0.81	1.08 ± 1.10	1.39 ± 1.23	**<0.001**	**<0.001**	**<0.001**
Cognitive problems	0.57 ± 0.75	0.59 ± 0.79	0.51 ± 0.58	0.207	0.192	0.206
Weight problems	0.48 ± 0.67	0.48 ± 0.93	0.56 ± 0.85	0.230	0.229	0.223
Arm problems	0.21 ± 0.50	0.14 ± 0.35	0.08 ± 0.28	0.643	0.661	0.709
WOMAC						
Total	11.7 ± 18.4	12.1 ± 18.4	14.3 ± 19.1	0.324	0.332	0.335
Pain	8.0 ± 14.6	11.2 ± 18.7	12.3 ± 17.6	**0.058**	**0.045**	**0.047**
Stiffness	17.9 ± 20.5	21.0 ± 22.9	27.1 ± 29.4	**0.028**	**0.031**	**0.031**
Physical function	12.0 ± 21.3	11.4 ± 18.4	13.4 ± 19.5	0.662	0.677	0.679
PHQ-9						
Total	3.61 ± 4.65	2.73 ± 2.88	2.46 ± 2.80	0.212	0.171	0.176

^a^ Mixed-effects model with time (date) as a continuous variable adjusted for baseline symptom score and clustered on the patient (no adjustments for multiple comparisons). ^b^ Further adjusted for age at baseline and BMI at baseline. ^c^ Further adjusted for definitive therapy (mastectomy vs. lumpectomy), Bolded are represent the significant *p*-values.

## Data Availability

The data presented in this study are available on request from the corresponding author. The data are not publicly available due to containing information that could compromise the privacy of research participants.

## Supplementary Materials

The following supporting information can be downloaded at: https://www.mdpi.com/article/10.3390/metabo13030452/s1, Table S1: Polyunsaturated Fatty Acid (PUFA) levels across time (ng/mL plasma), mean ± SD; Table S2: Oxylipin levels across time (pg/mL plasma), mean ± SD.
