# Supplementary material for: Oxylipins as Biomarkers for Aromatase Inhibitor-Induced Arthralgia (AIA) in Breast Cancer Patients
Source: Metabolites. 2023 Mar 20;13(3):452. doi: 10.3390/metabo13030452 (PMC10052117; doi:10.3390/metabo13030452)
Supplement: Supplementary file 1 [file metabolites-13-00452-s001.zip › metabolites-2285057-supplementary.pdf]

**Supplemental Table S1. Polyunsaturated Fatty Acid (PUFA) levels across time (ng/mL plasma), mean ± SD**				
**PUFA**	**Baseline** ***n* = 25**	**3 months** ***n* = 22**	**6 months** ***n* = 24**	***P*[a]**
Arachidonic acid (ARA)	524.3 ± 498.3	430.2 ± 210.0	452.7 ± 232.0	0.529
Linoleic acid (LA)	2,300 ± 2,300	2,300 ± 1,800	2,400 ± 2,200	0.538
Eicosapentaenoic acid (EPA)	220.4 ± 300.3	178.6 ± 245.3	155.6 ± 155.7	0.894
Docosahexaenoic acid (DHA)	4.5 ± 6.6	4.2 ± 5.6	4.1 ± 5.4	0.938

[a] Mixed-effects model of log-transformed PUFA level, with time (date) as a continuous variable, adjusted for batch and baseline oxylipin level, clustered on patient (no adjustments for multiple comparisons)

Supplemental Table S2. Oxylipin levels across time (pg/mL plasma), mean ± SD						
**Oxylipin**	**PUFA**	**Pathway**	**Baseline** ***n* = 25**	**3 months** ***n* = 22**	**6 months** ***n* = 24**	***P*[a]**
PGD_2	ARA	COX-2	5.4 ± 4.8	6.0 ± 5.6	5.8 ± 5.5	0.294
11-HETE	ARA	LOX	55.6 ± 45.6	59.3 ± 35.6	54.9 ± 43.7	0.787
12-HETE	ARA	12-LOX	237.7 ± 316.7	253.0 ± 309.3	482.8 ± 1,253	0.568
12-oxo-ETE	ARA	LOX	108.3 ± 94.1	141.7 ± 118.2	128.3 ± 106.1	0.536
5-HETE	ARA	5-LOX	103.3 ± 136.7	116.4 ± 103.1	100.6 ± 82.0	0.460
8-HETE	ARA	15-LOX	10.2 ± 5.9	12.1 ± 7.8	12.3 ± 9.8	0.684
15-HETE	ARA	15-LOX	115.3 ± 72.0	123.9 ± 66.8	149.2 ± 122.2	0.238
20HETE	ARA	CYP450	80.5 ± 50.0	81.5 ± 39.2	103.7 ± 96.9	0.076
5(6)-EET	ARA	CYP450	1,053 ± 1,354	2,096 ± 2,855	1,796 ± 2,526	0.105
8(9)-EET	ARA	CYP450	679.8 ± 1,041	1,311 ± 2,216	1,147 ± 1,913	0.472
11(12)-EET	ARA	CYP450	797.9 ± 798.4	1,356 ± 1,815	1,353 ± 1,794	0.070
14(15)-EET	ARA	CYP450	2,102 ± 2,646	3,922 ± 5,195	3,696 ± 4,768	0.058
5(6)-DiHET	ARA	sEH	25.3 ± 16.6	21.8 ± 10.9	30.2 ± 43.4	0.773
8(9)-DiHET	ARA	sEH	58.1 ± 64.4	58.4 ± 86.2	62.7 ± 71.7	0.850
11(12)-DiHET	ARA	sEH	116.3 ± 86.9	115.8 ± 66.9	140.8 ± 122.2	0.229
14(15)-DiHET	ARA	sEH	94.9 ± 74.8	104.2 ± 60.3	136.9 ± 199.2	0.126
9-HODE	LA	LOX	1,539 ± 2,063	1,761 ± 2,438	1,534 ± 1,801	0.795
13-HODE	LA	LOX	2,018 ± 2,440	2,180 ± 2,876	2,073 ± 2,267	0.776
13(S)-HOTrE	LA	15-LOX	141.8 ± 127.6	126.6 ± 87.6	106.4 ± 66.3	0.297
9-OxoODE	LA	15-LOX	113.9 ± 91.1	142.0 ± 98.9	136.2 ± 134.9	0.942
13-OxoODE	LA	LOX	245.0 ± 345.2	914.8 ± 2,331	286.6 ± 521.7	0.963
9(10)-EpOME	LA	CYP450	24,719 ± 46,172	36,935 ± 60,993	43,689 ± 73,959	0.072
12(13)-EpOME	LA	CYP450	17,522 ± 31,024	24,794 ± 36,137	32,565 ± 54,680	0.069
9(10)-DiHOME	LA	sEH	1,031 ± 1,543	1,297 ± 2,010	1,336 ± 1,497	0.068
12(13)-DiHOME	LA	sEH	1,458, ± 2,167	2,012 ± 2,858	1,851 ± 2,722	0.125
9-HOTrE	ALA	5-LOX	60.8 ± 41.6	62.1 ± 35.0	51.8 ± 34.9	0.500
7(8)-EpDPA	DHA	CYP450	572.9 ± 757.3	821.9 ± 1,225	940.8 ± 1,360	0.066
10(11)-EpDPA	DHA	CYP450	266.2 ± 506.8	464.8 ± 728.5	466.7 ± 859.7	0.441
13(14)-EpDPA	DHA	CYP450	219.7 ± 364.1	470.3 ± 808.5	369.8 ± 706.7	0.100
16(17)-EpDPA	DHA	CYP450	366.6 ± 617.5	386.7 ± 535.6	377.6 ± 653.3	0.616
19(20)-EpDPA	DHA	CYP450	1,005 ± 1,917	1,073 ± 1,602	1,534 ± 2,404	0.108
19(20)-DiHDPA	DHA	sEH	316.7 ± 243.8	348.8 ± 238.5	466.2 ± 707.4	0.163
5-HEPE	EPA	5-LOX	25.5 ± 27.7	31.3 ± 32.4	27.1 ± 21.2	0.581
8-HEPE	EPA	LOX	11.7 ± 18.5	13.8 ± 20.4	9.3 ± 5.7	0.827
12-HEPE	EPA	12-LOX	23.3 ± 68.5	17.2 ± 39.7	65.3 ± 173.5	0.173
15-HEPE	EPA	15-LOX	9.4 ± 8.8	9.0 ± 7.7	8.4 ± 9.5	0.386
8(9)-EpETE	EPA	CYP450	15.8 ± 19.3	49.2 ± 131.0	33.8 ± 57.4	**0.039**
14(15)-EpETE	EPA	CYP450	47.2 ± 60.4	160.9 ± 399.6	98.3 ± 191.9	0.083
17(18)-EpETE	EPA	CYP450	70.4 ± 70.4	250.6 ± 593.1	159.2 ± 325.0	0.111
5(15)-DiHETE	EPA	sEH	7.3 ± 14.3	20.8 ± 59.1	7.7 ± 17.4	0.953
8(15)-DiHETE	EPA	sEH	9.0 ± 21.8	10.8 ± 19.6	15.6 ± 20.6	**0.025**
14(15)-DiHETE	EPA	sEH	6.9 ± 5.9	5.8 ± 4.1	6.9 ± 6.9	0.766
17(18)-DiHETE	EPA	sEH	88.3 ± 78.3	88.5 ± 71.6	90.7 ± 72.1	0.852

[a] Mixed-effects model of log-transformed oxylipin level, with time (date) as a continuous variable, adjusted for batch and baseline oxylipin level, clustered on patient (no adjustments for multiple comparisons)

Abbreviations - ARA: Arachidonic Acid; LA: Linoleic Acid; ALA: Alpha Linolenic Acid; DHA: Docosahexaenoic acid; EPA: Eicosapentaenoic acid; COX: Cyclooxygenase; LOX: Lipoxygenase; CYP450: Cytochrome P450; sEH: soluble epoxide hydrolase
